# Highly Superior Autobiographical Memory: Quality and Quantity of Retention Over Time

**DOI:** 10.3389/fpsyg.2015.02017

**Published:** 2016-01-21

**Authors:** Aurora K. R. LePort, Shauna M. Stark, James L. McGaugh, Craig E. L. Stark

**Affiliations:** ^1^Department of Neurobiology and Behavior, University of California, Irvine, Irvine, CAUSA; ^2^Center for the Neurobiology of Learning and Memory, Department of Education, University of California, Irvine, Irvine, CAUSA

**Keywords:** Highly Superior Autobiographical Memory, autobiographical memory, obsessive–compulsive disorder, memory retrieval, recollection, human behavior

## Abstract

Individuals who have Highly Superior Autobiographical Memory (HSAM) are able to recall, with considerable accuracy, details of daily experiences that occurred over many previous decades. The present study parametrically investigates the quantity and quality of details of autobiographical memories acquired 1-week, 1-month, 1-year, and 10-years prior in HSAMs and controls. In addition, we tested the consistency of details provided at the 1-week delay by testing the subjects 1 month later with a surprise assessment. At the 1-week delay, HSAMs and controls recalled an equivalent number of events. In contrast, HSAM recall performance was superior at more remote delays, with remarkable consistency following a 1-month delay. Further, we revealed a relationship between the consistency of recall and HSAMs’ obsessive–compulsive tendencies. These data suggest that HSAMs experience normal encoding, yet enhanced consolidation and later recall of autobiographical events.

## Introduction

Individuals who have Highly Superior Autobiographical Memory (HSAM) demonstrate the ability to recall accurately vast amounts of remote salient autobiographical events without the explicit use of mnemonics (Parker et al., 2006; LePort et al., 2012). HSAM is readily distinguishable from other forms of exceptional memory such as that found in mnemonists. One technique for producing strong memories is through overt intensive memorization of material and or use of mnemonic techniques (Luria, 1968; Hunt and Love, 1972; Ericsson et al., 2004; Foer, 2011). In contrast, HSAM individuals report that they do not rehearse their experiences or use mnemonic techniques with the explicit intent to create strong memories, unlike many memory experts (Ericsson and Moxley, 2014). Interestingly, although they have exceptional autobiographical memory, they are no better than control subjects at laboratory memorization tasks (Parker et al., 2006; LePort et al., 2012). Therefore, the study of individuals who have strong and lasting memories of ordinary daily experiences provides a novel perspective from which to investigate memory encoding, storage, and retrieval.

Highly Superior Autobiographical Memory participants have a remarkable ability to recall details of personal and public events over an extensive range of lifetime periods (Parker et al., 2006; LePort et al., 2012). This ability suggests that they may be unable to forget and, thus, are able to preserve a remarkable richness of detail concerning autobiographical events. Normally, the passage of time is known to have two hallmark effects on event memory. First, forgetting of information tends to occur at a roughly exponential rate (Ebbinghaus, 1885; Wixted and Carpenter, 2007). Second, the quality of a memory, even the most memorable, generally becomes altered as time passes (Christianson, 1989; Levine et al., 2002). While individuals with HSAM have detailed memory for previous autobiographical events, the rate of forgetting and the quality and quantity of these memories has not been previously investigated in sufficient detail.

The goal of the present study was to further evaluate the number and accuracy of details retained over time. Do individuals with HSAM encode the same number of details as controls? Do they lose any details of their autobiographical memories over time? Does the quality of these memories decline over time? Are they susceptible to memory errors or distortions for these autobiographical events? We administered two autobiographical tests that asked participants to recall autobiographical information from every day in a specified week. In the first test session, both recent (each day of the past week) and remote (those same seven dates that occurred 1-year and 10-years ago). The second was a surprise 1-month follow-up test in which the most recent of those same dates were probed (allowing us to assess the consistency of the recalled information).

In addition, we explored the possibility that HSAM subjects’ obsessive tendencies may contribute to their extraordinary memory ability. Previously, we reported that HSAM participants’ display obsessional tendencies that may include the rumination of their past experiences (LePort et al., 2012). This frequent, possibly habitual, rumination may serve as a subtle, implicit rehearsal of autobiographical material, aiding in retention of details and consolidation into long-term storage. To further address this issue, we examined whether HSAM subjects’ ability to recall autobiographical details was correlated with the degree to which they display obsessive–compulsive tendencies. Thus, HSAM participants completed the Leyton Obsessional Inventory (Cooper, 1970) to evaluate their obsessional tendencies, which may contribute to the persistent rumination supporting these long-lasting memories.

## Materials and Methods

### Participants

A multi-step, Institutional Review Board (IRB) approved process was developed to identify and test HSAM and control participants. Individuals who contacted us proclaiming to have HSAM were screened using the Public Events Quiz and 10 Dates Quiz (LePort et al., 2012). Briefly, the Public Events Quiz contained two types of questions: half asked for the date of a given significant public event that took place within the individuals’ lifetime (e.g., When did Jimmy Carter win the Nobel Peace prize?) and the other half asked for the significant public event that took place on a given date that fell within the individual’s lifetime. In addition, for all 30 questions, individuals were asked to state the day of the week the date fell on. The order of presentation of the two types of questions was interchanged. Participants receive one point for each correctly identified category (i.e., the event, the day of the week, the month, the date and the year) and could achieve a total of 88 possible points. A score of 50% or above qualified an individual claiming to have HSAM to advance to the second, even more challenging round of screening, the 10 Dates Quiz (LePort et al., 2012).

The 10 Dates Quiz consisted of ten computer generated random dates, ranging from the individuals’ age of fifteen to the day of testing. Individuals were asked to provide three different categories of information for each of the 10 dates generated: (1) the day of the week; (2) a description of a verifiable event (i.e., any event that could be confirmed via a search engine) that occurred within ±1-month of the generated date; and (3) a description of a personal autobiographical event. One point was awarded for the correct day of the week, for giving a verifiable event confirmed as true, and/or for giving a personal autobiographical event. A maximum of three possible points per date could be achieved (30 points total). The percentage scored for each category as well as the total score, the average of all three categories, was calculated. A total score of 65% or above qualified the individual as an HSAM participant.

Following the screening procedure, 30 HSAM participants (24 males; mean age: 39 years old, range: 19–68) were tested remotely via telephone. In addition, 20 controls (11 male; mean age: 42 years old, range: 22–65) were recruited from the community and tested in the same manner. Two HSAM participants and one control participant failed to complete the initial portion of the study; An additional HSAM and nine additional control participants failed to complete the 1-month retest portion of the battery, due to an inability to get in touch with them. All participants were compensated monetarily for their participation. All independent variables or manipulations and all dependent variables or measures, analyzed for this article’s target research questions, are reported in the section “Materials and Methods” section. This study was carried out in accordance with the recommendations of the University of California at Irvine IRB with written consent from all subjects. All subjects gave written informed consent in accordance with the Declaration of Helsinki.

Since there is very little prior research investigating HSAM, it is difficult to accurately estimate effect sizes and thereby calculate the sample size needed to detect group effects. One study assessing autobiographical memory in major depressive disorder reported an effect size of *d* = 1.00 concerning the number of autobiographical details patients recalled as compared to controls over time (Soderlund et al., 2014). A study assessing the number of autobiographical events from various time points that individuals with ‘time-space’ synesthesia could recall, as compared to controls, reported an effect size of *d* = 1.707 (Simner et al., 2009). Cautiously, a medium effect size is assumed to detect potential differences between our groups. Assuming an effect size of *d* = 0.40, α = 0.05, and 1–β = 0.80, a total sample size of 52 participants was calculated using *G∗Power 3.1.9.2* (Faul et al., 2009).

### Procedure

In a structured interview, participants were asked to give as many details as they were comfortable reporting for particular dates in question. They were told not to gloss over routine or repetitive events and to only report details of events they were able to recall. They were instructed to be chronological, commencing with waking in the morning. To give participants a clear understanding of the level of detail desired, a practice trial was conducted in which participants reported details of the present day. Participants were probed, only for this exemplar, until the participant attained an idea of the level of detail to include. For instance, if a participant stated during the practice trial that they ate breakfast they would be probed as to what they specifically ate. During test, participants were uninterrupted. At the completion of each date they were asked if they had been as descriptive as possible. In order to guard against fatigue, a two-min time limit was set for each date. If they did not reach the two-min time limit they were probed once, at the completion of each date, with the question, “is there anything else you can recall?” Two HSAM participants and one control did not deliver a level of detail that they were able to, as they stated they did not wish to share the information. Consequently, these participants were eliminated from the study.

Two autobiographical memory tests were administered remotely via telephone and all responses were recorded and later transcribed. The first test queried recent dates, each day of the past week, as well as remote dates; those same seven dates that occurred 1-year and 10-years ago. The second was a surprise 1-month follow-up test in which the most recent of those same dates were probed (allowing us to assess the consistency of the recalled information). For example, if a participant was scheduled for the first test on April 16, 2014, he or she would have been asked about the events of April 15-9 of 2014, 2013, and 2004. Approximately on May 16, 2014, the participant would again be asked about the events of April 15-9 of 2014. Given the 2-min time limit, it took a maximum of 42 min to complete all dates in the first interview and a maximum of 14 min to complete the second test, given 1-month later.

For only the most recent seven dates, almost all HSAM and control participants had more to report than could be recorded within the 2-min time limit. Both groups completed all other time points (1 month, 1 year, and 10 years) in the allotted time. To assess ceiling effects imposed by the time limit, five previously tested HSAM participants and five controls were retested (following the same guidelines) with no time restriction, after completion of the original study. Only two dates were queried: 1 day prior and 6 days prior to the test date (e.g., if the retest date was June 16, 2014, the test dates would be June 15_,_ 2014 and June 10, 2014).

### Data Analysis

The data were analyzed in several ways. The first measured quantity and quality of details HSAM participants and age- and sex-matched controls reported. Data at each delay time point (1-week, 1-month, 1-year, 10-years) were averaged over the week. The second observed the rate at which participants forgot details over the most recently occurring, week. The third evaluated the rate at which the quality of details changed over the most recently occurring week. The fourth measured the quantity and quality of details participants, who were retested, reported from 1-day and 6-days ago (without being restricted by a 2-min time limit). The fifth, by means of the Layton Obsessional Inventory (LOI), surveyed the degree to which participants express obsessional symptoms/tendencies. Lastly, a correlation was made between the measured obsessional behaviors and how consistent details remained from the 1-week to retest at the 1-month time point.

### Quantity

Details were divided into internal and external details. Each detail was counted and summed, providing the total number of internal or external details given per date. An internal detail was defined as, “a unique occurrence, observation, or thought” as defined in the Autobiographical Memory Interview (Levine et al., 2002). For instance, “I took my brother to the dentist in the morning” contains three details: brother, dentist, and morning. Only details that described the event in question were included in the total internal score. External details were defined as details not specific to the day in question, repetitions, metacognitive statements, and editorializing (Levine et al., 2002). In addition, a ratio of total internal to total internal plus total external details was calculated.

### Quality

To distinguish the quality of each internal detail, they were grouped into events. An event had its own story line and was distinguished by its unique subject, time, place, and descriptors. Two broad groups were made, categorizing details into “gist” and “peripheral” elements. Using a categorization technique typical to the field (Heuer and Reisberg, 1990; Cahill and van Stegeren, 2003), gist information was defined as, “information that cannot be removed or altered without changing the fundamental story line.” All other details were defined as pertaining to peripheral information. External details were not categorized into gist and peripheral details and were not included in the total internal quality score because they did not reflect autobiographical information. In the aforementioned example, “brother” and “dentist” would be considered gist details, whereas “morning” would be considered a peripheral detail.

To reduce biases during scoring, the quantity (sum of internal gist and internal detail elements) and quality (categorization into internal gist vs. internal peripheral elements) of all dates were determined separately by two independent scorers (grader A and grader B) blind to the other’s scores. An inter-rater reliability score between 0.8 and 1.0 indicated high agreement and was a necessary criterion for the inclusion of the date in the study. Inter-rater reliability was calculated by subtracting from 1, the absolute value of the quantity, quality detail or quality gist score of grader A divided by the total (i.e., graders A+B) quantity, quality detail or quality gist score – quantity, quality detail or quality gist score of grader B divided by total quantity, quality detail or quality gist score. The average inter-rater reliability scores were: 0.96, 0.92, and 0.92, respectively. No dates were excluded. Finally, percentage scores for each class (gist vs. peripheral) were computed as their respective portions of the total number of internal details (gist plus peripheral).

### Consistency

In order to verify the consistency of details across time, 1-month later, participants were queried about the same dates from the original week. Loosely modeling criteria developed by (Christianson, 1989), a detail was determined to be consistent if it was an equivalent detail, though not necessarily the same word. A detail was considered inconsistent (i.e., incorrect) if it was clearly referring to an event shared during the original week, but was not the equivalent detail. Details unrelated to the original event (i.e., new details) shared at the 1-month time point were not included in the analysis; Only a portion of the potential information from the days of the original week was attained, due to the 2-min time limit. Therefore new details could be from events that had occurred 1-month prior, but were not shared originally. New details were unverifiable and were therefore *not* considered incorrect. They were not included in the analysis. Consistent details were categorized into gist and peripheral elements. “Percent consistency” was calculated by dividing total gist and peripheral consistent details by total details recalled from the original week. An inter-rater score between 0.8 and 1.0 indicated high agreement. This score was a necessary criterion for the inclusion of the score in the study. All scores passed this criterion.

### Leyton Obsessional Inventory

Prior interviews with a number of HSAM individuals revealed a predisposition to recall and order events of their lives. Importantly, in our previous study, the LOI-Short Form (Mathews et al., 2004) was administered and results indicated that the 11 HSAM participants expressed significantly more obsessional tendencies than did their matched controls (LePort et al., 2012). Subjective assessments of obsessional traits and symptoms were therefore collected from 32 HSAM participants with the long-form version of the LOI. The complete LOI consists of 69 questions from which a “symptom score” was produced. Unlike the original protocol (Cooper, 1970), the survey questions were delivered with SurveyMonkey, an online survey system, rather than an in person interview.

## Results

### Overall Forgetting

**FIGURE 1:**
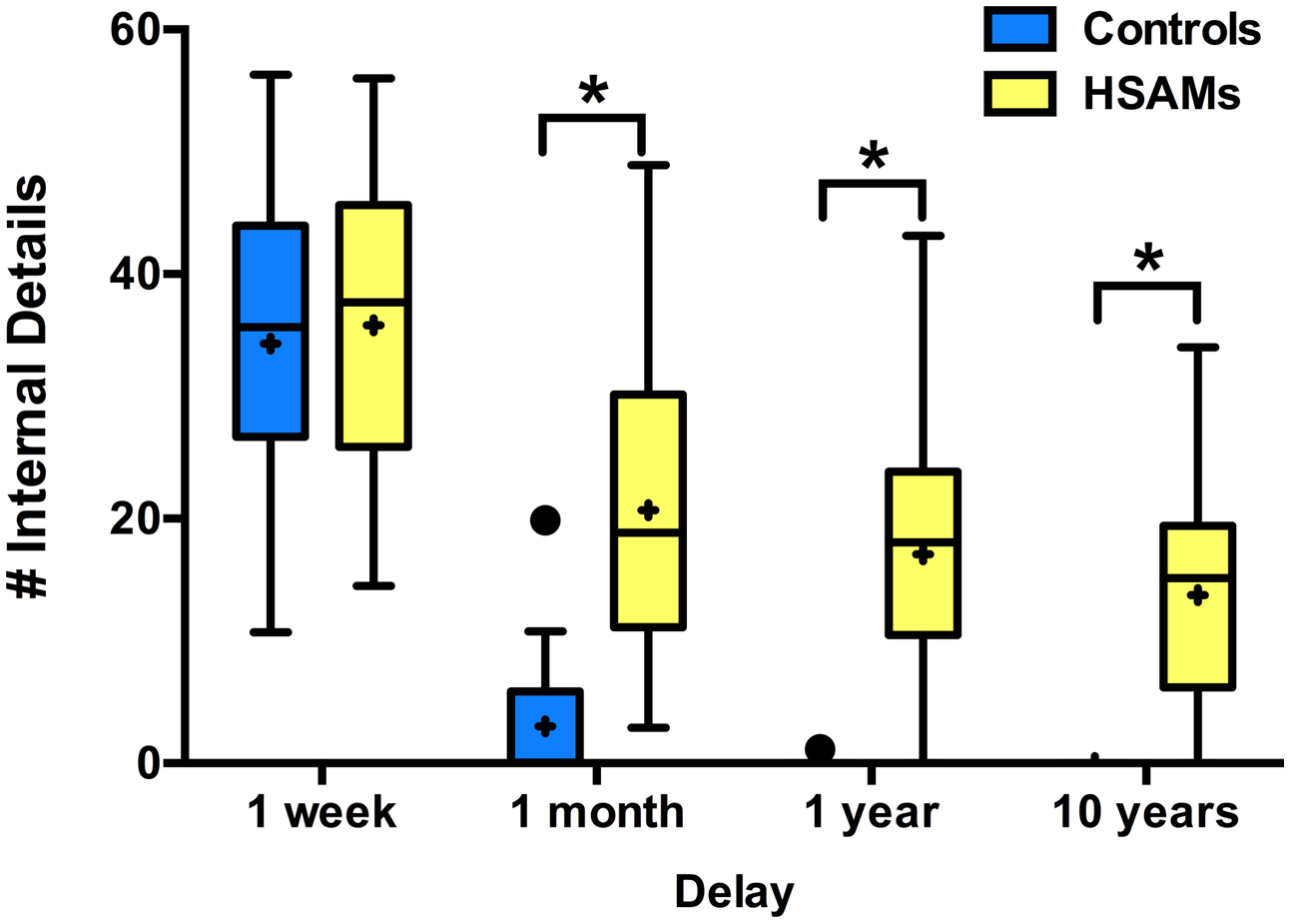
**Total # of internal details recalled over time.** Box and whisker plot (Tukey style) represents number of autobiographical details, averaged over 7 days, each group can recall at the four delay points (Highly Superior Autobiographical Memory; HSAM *n* = 27, yellow bars; control *n* = 20, blue bars). HSAM participants recall significantly more details at the 1-month, 1-year, and 10-year time points. ∗*p* < 0.001. “+” indicates average value.

Highly Superior Autobiographical Memory and control participants recalled a similar number of internal details (**Figure 1**) from the 1-week period, but HSAM participants recalled significantly more details at the more remote time points (1-month, 1-year, and 10-years). The average number of details HSAM participants (*n* = 27) could recall from the average of the entire week for each of the four delay points was calculated and compared to that of the controls (*n* = 20) across time. A two-way repeated measures ANOVA of group (HSAM vs. Control) by delay (1-week, 1-month, 1-year, 10 years) revealed a significant main effects of group and delay [*F*_(1,45)_ = 39.82, *p* < 0.001, η_p_^2^= 0.50; *F*_(3,135)_ = 147.2, *p* < 0.001, η_p_^2^= 0.77] as well as a significant interaction (*F*_(3,135)_ = 12.06, *p* < 0.001, η_p_^2^= 0.21). Multiple comparisons-corrected (Sidak) analyses revealed significant group differences at the 1-month, 1-year, and 10-year delay points (*p* < 0.001, 95% CI: [10.83, 24.54], [10.14, 23.85], and [6.90, 20.67], respectively) excluding 1-week (*p* > 0.250, 95% CI: [–5.36, 8.36]). Notably, for HSAM participants, the number of internal details retrieved at the 1-week delay was significantly higher than that at the 1-month delay (*p* < 0.001, 95% CI: [9.77, 20.44]; Sidak correction for multiple comparisons). Interestingly, the number of details at the 1-week time point was comparable for HSAMs and controls, indicating comparable encoding of events. In contrast, while recall of later time points experienced decay over time in the HSAMs, it severely declined in controls.

**FIGURE 2:**
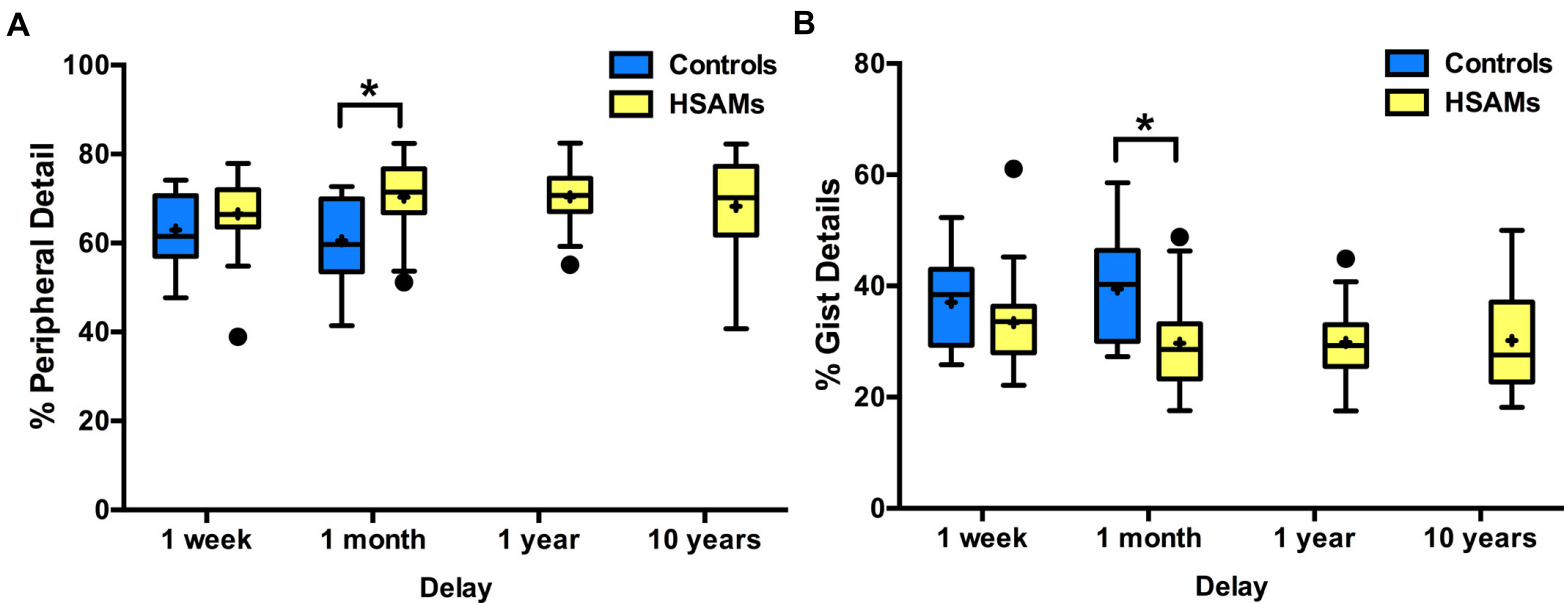
**Percentage internal peripheral and internal gist detail recalled over time.** Box and whisker plots (Tukey style) represent **(A)** percent peripheral details and **(B)** percent gist details recollected at each delay (HSAM *n* = 27, yellow bars; control *n* = 10, blue bars). HSAM participants recall significantly more gist and peripheral details at the 1-month time point. ∗*p* < 0.001. Graph B is (and must be) a mirror image of A. Control data is only represented for the 1-week and 1-month delays as there was insufficient data for 1-year and 10-year delays. “+” indicates average value.

We also calculated the average quality of internal details (%-peripheral and %-gist detail) that HSAM participants could recall from average weeks at each of the four delay points. HSAM participants recalled a similar percentage of peripheral/gist detail as controls at the 1-week time point (**Figure 2**), but a higher percentage of peripheral, lower percentage of gist, detail at the 1-month time point (as the two measures must mirror each other, we cannot ascribe the effect to one or the other and show both to remain unbiased). HSAM scores are represented for each of the four delays, while control scores are represented for only the 1-week and 1-month time points (these are the only time points that contained enough control data to provide valuable information). While all 27 HSAM participants could provide autobiographical details at the 1-month time point, only 10 of 20 controls were able to recall enough events. Neither HSAMs nor controls could provide information past the two-min time restriction and so none of the participants’ narratives were cut off at this time point. A two-way repeated measures ANOVA of group (HSAM, *n* = 27 vs. Control, *n* = 10) by delay (1-week and 1-month) revealed a significant interaction [*F*_(1,35)_ = 6.62, *p* = 0.010, η_p_^2^= 0.16] and significant main effect of group [*F*_(1,35)_ = 9.60, *p* = 0.003, η_p_^2^= 0.30], but no main effect of delay [*F*_(1,35)_ = 1.12, *p* > 0.250, η_p_^2^= 0.03]. Correcting for multiple comparisons, we found a significant difference at the 1-month time point (*p* < 0.001, 95% CI: [6.85, 24.82]; Sidak correction), but not at the 1-week (*p* > 0.250, 95% CI: [-5.78, 12.19]). While both HSAMs and controls were able to recall a comparable number of details and gist at 1-week, after 1-month, HSAMS could provide details whereas controls rely more heavily on gist information.

### Quantity Past 7 Days: Linear Regression

**FIGURE 3:**
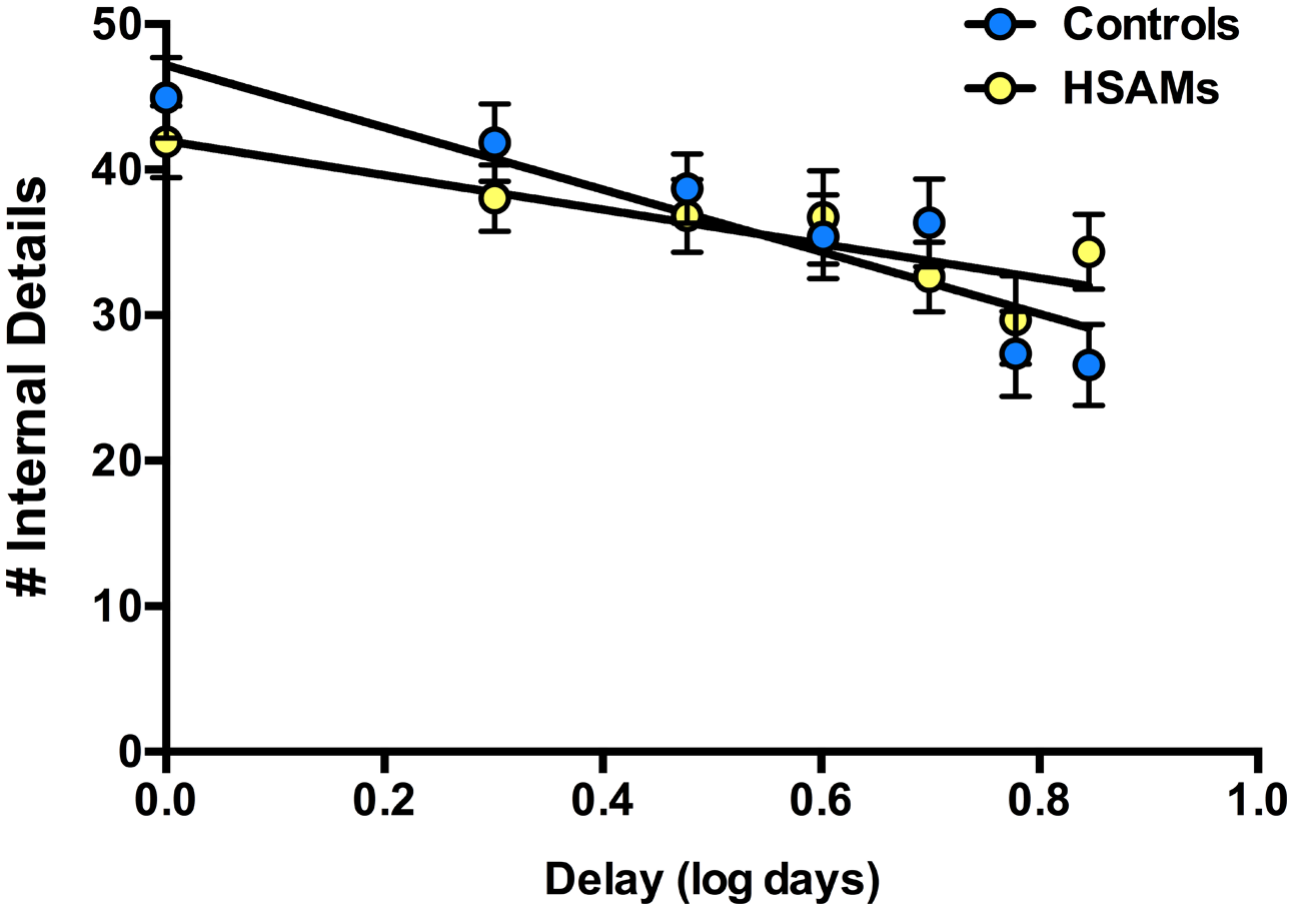
**Total internal autobiographical details recalled over past 7 days.** HSAM participants (*n* = 28, yellow circles); Controls (*n* = 29, blue circles). Slopes do not differ significantly from one another (*p* = 0.071). All values are means ± SEM.

To further examine the finding that HSAM and control memory was comparable when details were averaged over the first week, we broke the data down by day for a more detailed *post hoc* analysis (**Figure 3**). The overall forgetting rate is similar within the first week, albeit with some evidence for a more rapid forgetting in the controls. In this analysis, time delay values were log transformed to account for the typical shape of forgetting curves and thus better represents the data. Linear regression analyses revealed that although there was a trend toward a difference in the decay rates, it was not significant (*p* = 0.071, 95% CI [−21.84, −11.48], combined *r*^2^ = 0.09). Thus, the forgetting rate within the first week appears to be comparable for the two groups.

### Quality Past 7 Days: Linear Regression

**FIGURE 4:**
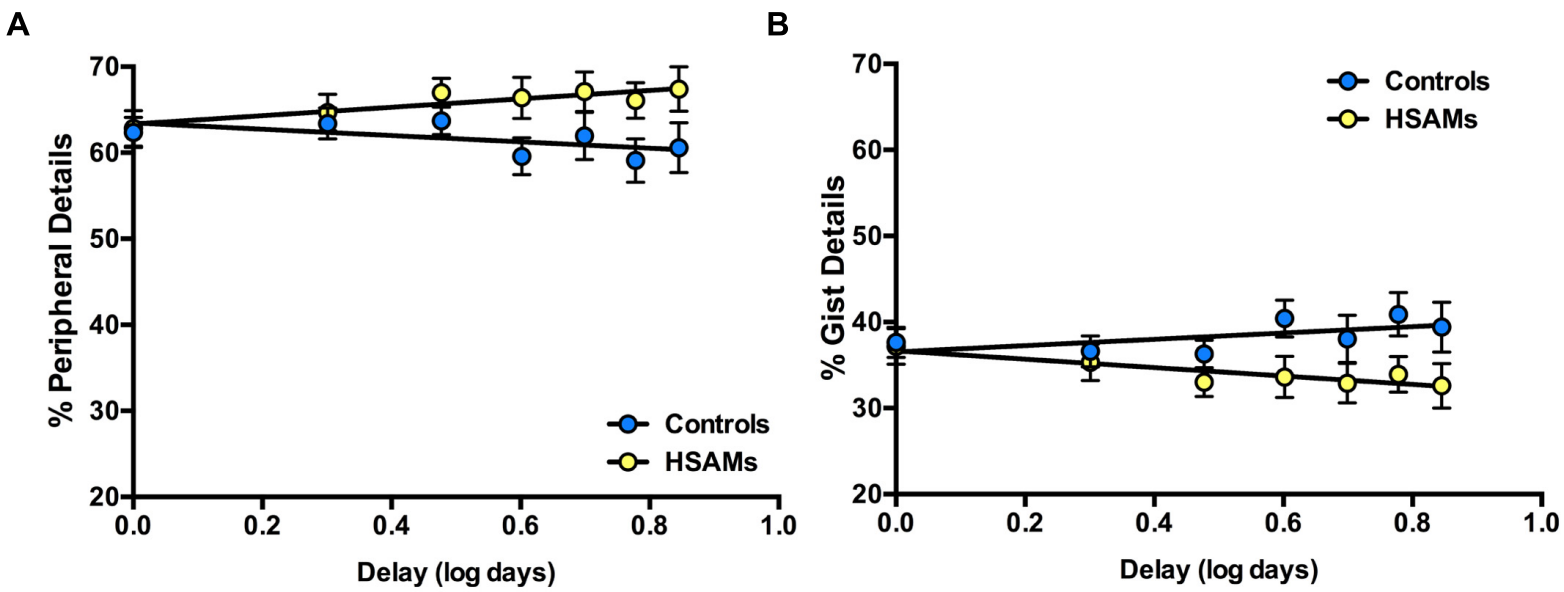
**Percentage of autobiographical peripheral and gist details recalled over past 7 days.** Log transformation of linear regression representing percentage of autobiographical peripheral and gist details recalled over the past 7 days. HSAM participants (*n* = 28, yellow circles); Controls (*n* = 29, blue circles). **(A)** HSAM participants’ recollections maintain a greater percentage of peripheral detail over time. **(B)** Control participants’ recollections maintain a greater percentage of gist detail over time. Slopes are significantly different from one another for graphs A and B (*p* = 0.046). Graph **(A)** is (and must be) a mirror image of **(B)**. All values are means ± SEM

We next turned to the question of the quality of memory during this first week by examining the proportion of internal gist vs. peripheral details in the participants’ recollections (and thus factoring out any potential differences in the number of recollections). As seen in **Figure 4**, of the details recalled, HSAM participants’ (*n* = 28, yellow circles) recollections over the past seven days were dominated more by peripheral details (and therefore less by gist details) than those of controls (*n* = 29, blue circles). As before, time delay values were log transformed to account for the typical shape of forgetting curves to better represent the data. Linear regression was used to determine that the slopes significantly differed (*p* = 0.046; 95% CI [−3.68, 4.75], combined *r*^2^ = 0.03), indicating that over the course of 1-week, HSAMs’ memory became less gist-based and contained proportionally more peripheral detail relative to controls’ recollections. Although the number of internal details was comparable over 1-week for the two groups (quantity), the level of details (detail vs. gist) differed between the two groups (quality).

### Quantity and Quality No Time Restriction

**FIGURE 5:**
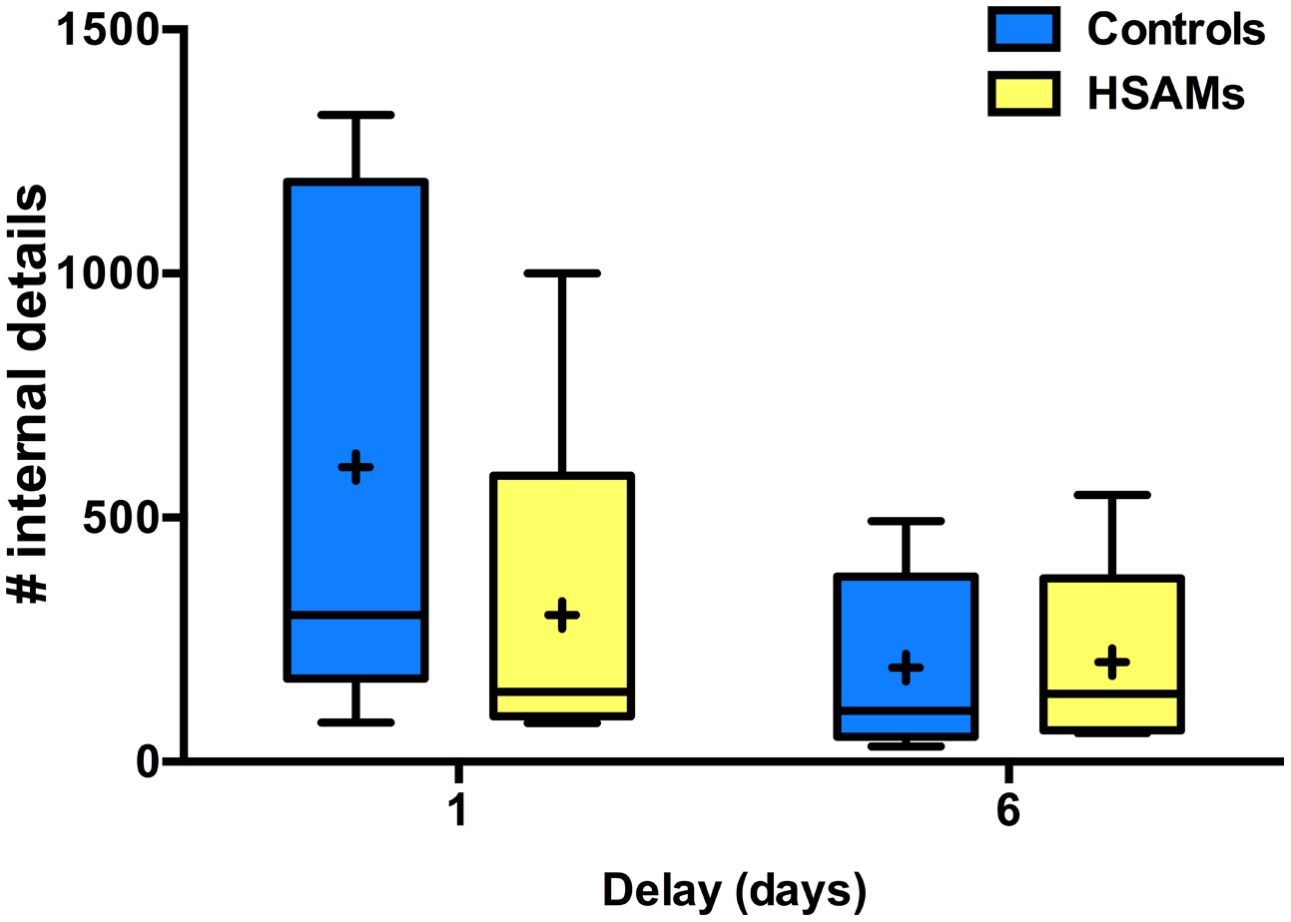
**Quantity of total internal autobiographical information recalled, no time restriction.** Box and whisker plot (Tukey style) per group (HSAM *n* = 5, yellow; control *n* = 5, blue) represents number of details recalled a 1 and 6 day delay. HSAM and control participants recall similar number of details at a both delays. “+” indicates average value.

A potential concern with the prior analyses is that participants were limited to 2 min of recollection for each probe date. We therefore retested in a subset of HSAM and control participants (*n* = 5 in each group) having them recall new dates occurring 1 and 6 days prior with no time limit. When this limit was removed, HSAM and control participants recalled a similar number of details from days 1 and 6 (**Figure 5**). A two-way repeated measures ANOVA of group (HSAM vs. Control) and delay (1 and 6 day) revealed no main effects of delay or group and no interaction on the number of total details recalled [*F*_(1,8)_ = 5.08, *p* = 0.054, η_p_^2^= 0.39; *F*_(1,8)_ = 0.53, *p* > 0.250, η_p_^2^= 0.17; *F*_(1,8)_ = 1.95, *p* = 0.201, η_p_^2^= 0.20]. Therefore, given an open response time, both groups were able to recall similar amounts of information for recent time points.

**FIGURE 6:**
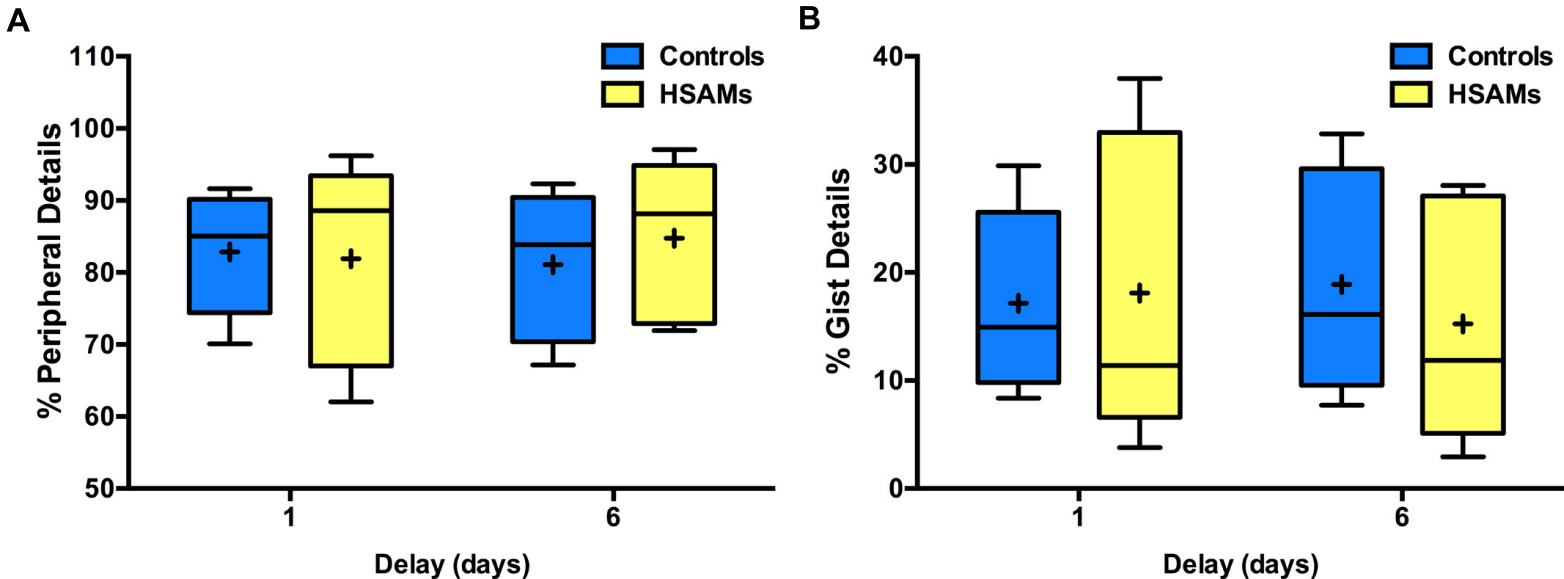
**Percent internal peripheral and gist detail, no time restriction.** Box and whisker plots (Tukey style) of **(A)** percent peripheral detail and **(B)** percent gist detail recalled (HSAM *n* = 5; control *n* = 5) at 1 and 6 day delays. HSAM and control groups recall similar percentage of gist and peripheral details at both delays. Graphs mirror each other. “+” indicates average value.

However, in contrast to data from the 2-min condition, HSAM and control participants’ memories were similarly composed of gist and peripheral details when the time limit was removed (**Figure 6**). In analyzing the percentage of gist (peripheral) details recalled, a two-way repeated measures ANOVA of group (HSAM vs. Control) and delay (1 and 6 day) revealed no main effects of delay or group and also no interaction [*F*_(1,8)_ = 0.07, *p* > 0.250, η_p_^2^= 0.01; *F*_(1,8)_ = 0.04, *p* > 0.250, η_p_^2^= 0.05; *F*_(1,8)_ = 1.21, *p* > 0.250, η_p_^2^= 0.13]. Thus, the two groups recalled comparable amounts of information when there was no time limit.

### Leyton Obsessional Inventory

**FIGURE 7:**
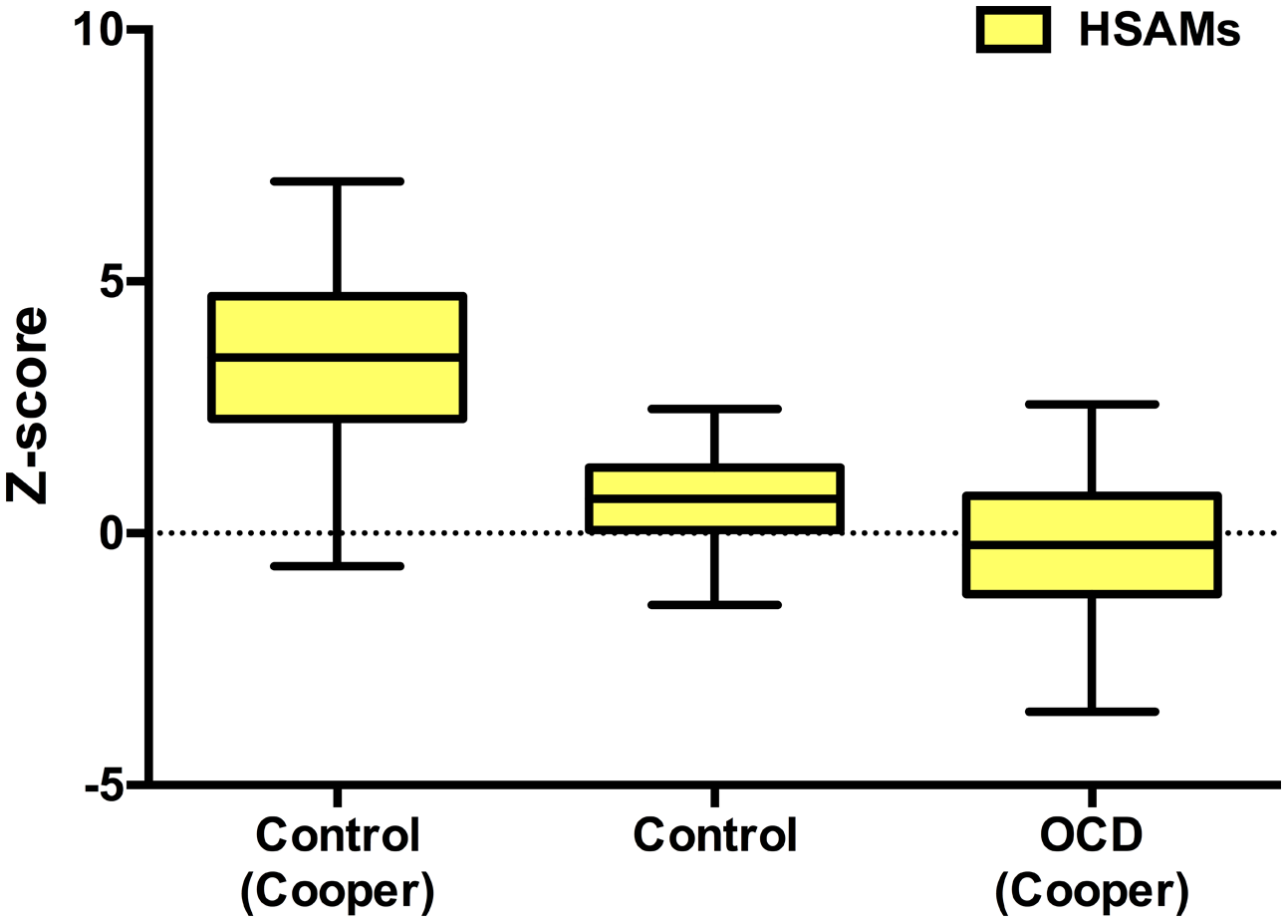
**Leyton Obsessional Inventory (LOI).** Box and whisker plot (Tukey style) representing HSAM symptom scores *z*-transformed and normalized to Cooper and our own control as well as Cooper OCD populations’ symptom scores. HSAM scores were reliably different from both control populations; ∗*p* < 0.001. HSAM and OCD patient population symptom scores were indistinguishable (*p* > 0.249).

To investigate obsessive-compulsive tendencies of HSAM subjects, their symptom scores (*n* = 32, *M* = 31.75, *SD* = 11.02) were normalized using *z*-scores to both a control (*n* = 101, *M* = 10.05, *SD* = 6.15) and an OCD population (*n* = 17, *M* = 33.3, *SD* = 7.7) using the normative data from Cooper (1970). They were also normalized to our own control population (*n* = 18, *M* = 23.22, *SD* = 12.08). Interestingly, LOI symptoms scores of our control population were significantly higher than that of Cooper’s controls. Nonetheless, HSAM participants’ symptom scores were reliably different from both Cooper’s and our own control population. **Figure 7** shows HSAM *z*-scores relative to the three populations. Significantly positive *z*-scores were found relative to both Cooper’s and our own control population (*M* = 3.53, *SD* = 1.79; *t*(31) = 11.13; *r* = 0.94; two-tailed Mann–Whitney, *p* < 0.001, 95% CI [2.88, 4.18] and *M* = 0.71, *SD* = 0.91; *t*(31) = 4.38, *r* = 0.60; two-tailed Mann–Whitney, *p* < 0.001, respectively, 95% CI [0.38, 1.04]). HSAM symptom scores were indistinguishable from the OCD patient population normative data, as evidenced by a *z*-score that did not reliably differ from zero (*M* = –0.20, *SD* = 1.43; *t*(31) = 0.80, *r* = 0.12; two-tailed Mann–Whitney, *p* > 0.250, 95% CI [-0.72, 0.32]). Consistent with our prior findings using the LOI-short form (LePort et al., 2012), HSAM participants display high obsessional tendencies, scoring in the range of an OCD population.

### Relationship of LOI to Consistent Autobiographical Information: Linear Regression

**FIGURE 8:**
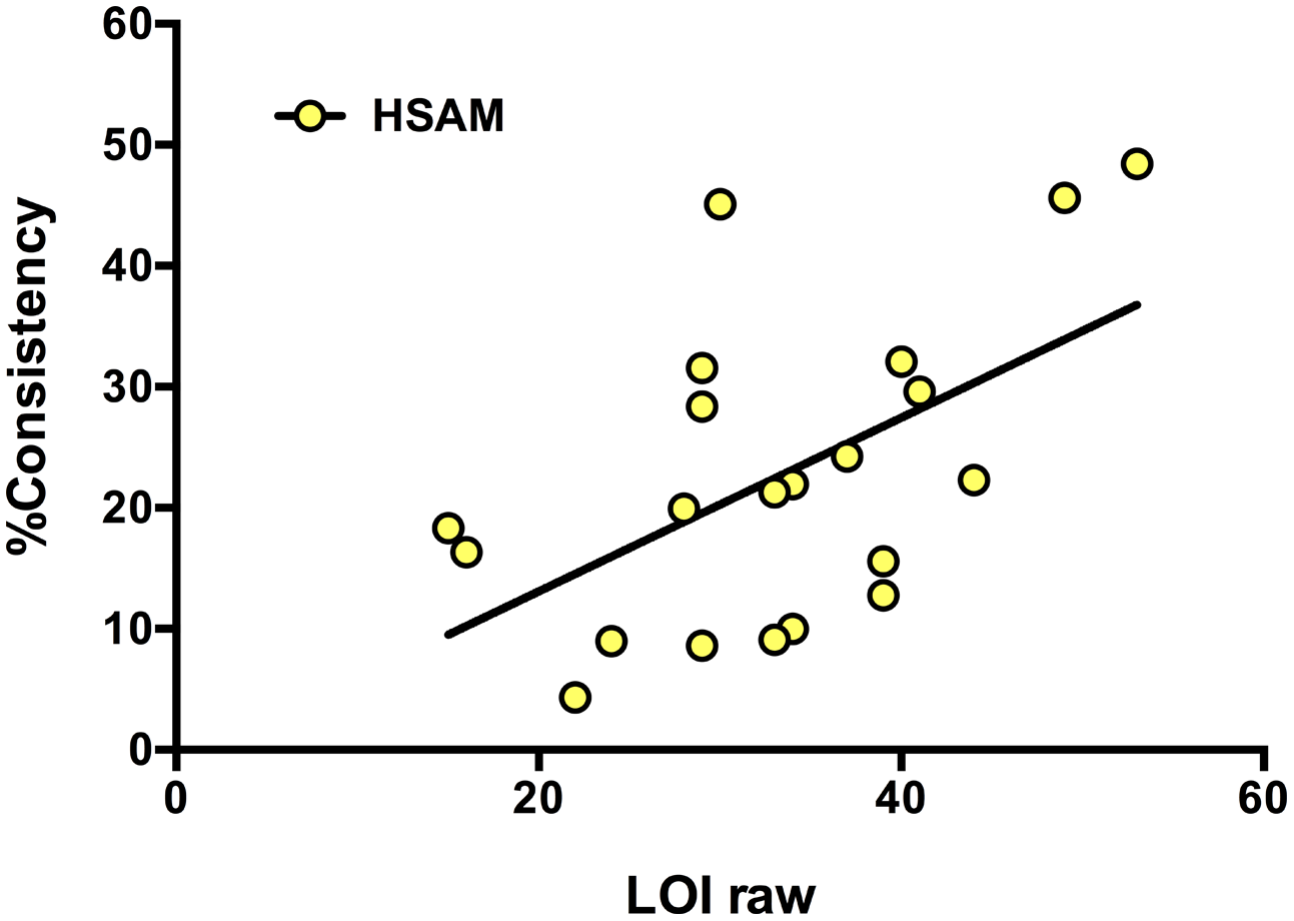
**Consistency.** Linear regression represents a significantly positive correlation between percent consistency of HSAM autobiographical details, from 1-week to 1-month time points, and symptom score on the LOI. HSAM participants (*n* = 19, yellow circles; *r*^2^ = 0.30, *p* = 0.010).

We then investigated the relationship between symptom scores and HSAM ability. LOI symptom scores were available for 19 of the HSAM participants. For these, we observed a correlation between their HSAM ability and their LOI (*p* = 0.010, 95% CI [01.9, 1.25], *r*^2^ = 0.30). HSAM participants who scored higher on the LOI tended to give more consistent details from autobiographical events tested and then retested 1-month later (**Figure 8**). In contrast, for a similar correlation was not seen in the controls who were able to report details at the 1-month time point (*n* = 6, *p* > 0.250, 95% CI [-2.44, 3.35], *r*^2^= 0.04). These data suggest that the obsessional tendencies exhibited by the HSAM group may contribute to their autobiographical memory recall.

## Discussion

We investigated recall of recent and remote, predominantly routine, events in HSAMs and controls. Interestingly, HSAMs and controls recall the same amount of information when tested within a few days (up to a week). Beyond this, HSAM participants forget autobiographical details at a far slower rate than do age- and sex-matched controls. Furthermore, in comparison with controls, HSAM participants maintain more richly detailed recollections and their forgetting curve for autobiographical memory is shallow, which should come as no surprise given the nature of their memory (LePort et al., 2012). The data here suggest that HSAMs are not better than controls in acquiring information. However, they are far superior at retaining information.

Over time, HSAM subjects continue to recall peripheral, whereas controls gradually rely more on gist details. HSAM participants’ exceptional ability to recall experiences (of mainly routine days) in rich detail was readily apparent at both the 1-year and 10-year time points, while control memory was nearly non-existent. Moreover, at the 1-month time point, control participants who could retrieve memories had significantly more generalized recollections than those of HSAM participants. This finding suggests that autobiographical memory in HSAM, particularly for mundane events, remains more “episodic-like” as time passes than it does for the rest of us. A strictly “episodic memory” encompasses perceptual, affective, and spatiotemporal contextual details derived from an event belonging to a specific instance in a rememberer’s personal past. It has been defined as an instance that one can mentally time travel back to and re-experience (Wheeler et al., 1997; Moscovitch et al., 2000). The richness of HSAM participants’ details is what makes their memory remarkable. In comparison, control subjects’ remote memories of ordinary daily experiences are either lost or are become so vague that they eventually represent generalized, or semanticized, experiences.

One inherent problem in studying autobiographical memory is the limited ability to verify the accuracy of the recalled material. The consistency of the recalled information from 1-week to 1-month lends some credibility that the events recalled truly occurred. A concern might arise that the HSAM individuals might invent information to remain consistent with their status as having HSAM. We find this unlikely given that they recalled equivalent amounts of information as controls during both in the time-limited and unlimited sessions. If they were confabulating to inflate their HSAM status, one would imagine that they would provide more overall information than controls during the unlimited recall sessions, yet we find no such evidence. In addition, the details that they repeated were remarkably consistent across sessions, though they tended to also add new information, indicating that these details were based on actual experiences. Finally, our prior work has demonstrated that when the events can be checked, they are exceptionally accurate (Parker et al., 2006; LePort et al., 2012)

The difference in the memory of HSAMs and controls may be discernable early on as the HSAM participants appear to emphasize details differently from controls when response time is constrained. At day six, HSAM participants reported more detailed information than controls given a 2-min time limit restriction. However the two groups did not differ in the number of details reported when there was no time restriction. This difference may reflect HSAM subjects’ expertise in recollecting autobiographical information. HSAM participants are experts in recalling their personal narrative. As result, they may have a sense of which bits of information are most relevant to maintain a truly episodic account of their life. When pressed for time, HSAMs may report more episodic-like details because they are readily available. The fact that 1-month later HSAM participants are still structuring information in a similar way supports this notion. As described above, 1 month after the original testing, their narrative had lost certain elements. Yet the bits of information that upheld the episodic nature of the memory were preserved, leaving their narrative rich in detail, while the controls’ had become more generalized. It is worth noting that the “no time restriction” data were acquired from a limited number of participants. Future studies interested in analyzing the quantity and quality of details retrieved by HSAM participants under these conditions would benefit from a greater sample size.

Thus, HSAM participants do not appear to be superior learners or display enhanced encoding. When tested 1 day after encoding an experience, the retention performance of HSAM subjects did not differ from that of controls; neither the number of details recalled nor their nature (gist vs. peripheral) differed from that of controls. Although HSAM participants maintain and retrieve a significant quantity of autobiographical information over long intervals of time, they do not excel at encoding either autobiographical or standardized cognitive information (LePort et al., 2012). Our previous finding that false memories occur at the same rate in HSAM participants as compared to controls is also consistent with this finding (Patihis et al., 2013). The fact that HSAM participants do not flawlessly encode information increases the likelihood of errors being introduced as memories are reconstructed.

Our findings suggest that HSAM may well be the result of the more efficient consolidation and retrieval of these detailed memories, perhaps rooted in obsessively driven, habitual rehearsal of autobiographical material. The obsessive–compulsive assessment (LOI) suggests that HSAM participants have obsessive behaviors (rumination, need for organization in their environment, germaphobia) similar to that of patients diagnosed with obsessive–compulsive disorder (LePort et al., 2012). It is known that memory can be significantly strengthened by distributed study and active retrieval (Roediger and Karpicke, 2006; Schwartz et al., 2011; Karpicke, 2012). Therefore, routine ruminations/perseverations of autobiographical information may serve to preserve HSAM participants’ memories. In fact, participants have reported that they sometimes think about what occurred on this day and compare it to what occurred 1 or 5 years earlier to lull themselves to sleep or when stuck in traffic. However, the available evidence does not support an interpretation that HSAM relies on explicit rehearsal or a deliberate strategy. What sets HSAM participants apart is that when they choose to ruminate, they can recall what happened on specific days from long ago. If this ability were due entirely to explicit rehearsal, achieving HSAM would require as much devotion to memorizing life events as a world memory champion devotes to memorizing, for example, decks of cards (Foer, 2011). As routines and pressures of daily life demands seem to bar the typical HSAM participant from this level of commitment to the maintenance of their memories, the strengthening may involve passive rumination without the intent of improving memory.

Rather, we suggest the possibility that HSAM participants may be *incidentally* strengthening their memories. In fact, it may be that HSAM is a unique form of OCD. Not only do HSAM participants express obsessive tendencies, but also we have found previously similarities between the structure of their brain and that of OCD patients. Namely, both populations share an enlarged caudate and putamen (LePort et al., 2012). It is worth noting that many OCD patients actually demonstrate impairments in autobiographical memory, which may be a result of co-morbid diagnosis of depression (Wilhelm et al., 2011). In contrast, HSAM participants, by means of an obsessive habit, may acquire and habitually use an implicit ability to embed autobiographical information within a larger memory network.

Subsequent research clarifying neurobiological similarities and differences between an OCD patient population and HSAM participants would provide further insight into mechanisms of HSAM more greatly related to OCD or autobiographical memory. Namely, similarities in the functional connectivity of the orbitofronto-striatal circuitry would signify a pathophysiology comparable to that of OCD patients (Menzies et al., 2008). Differences in the functional connectivity of the Default Mode Network, previously shown to be important for self-referential thought, self-projection and autobiographical remembering (Buckner et al., 2008), would point to neurobiological processes possibly contributing to HSAM, but distinct from OCD. Future research will be needed to directly compare the neural and behavioral profiles of HSAM and OCD to further investigate this relationship.

## Author Contributions

AL, SS, CS, and JM are the authors of this article and are responsible for its content. All authors developed the study concept and contributed to the study design. Testing, data collection, data analysis were performed by AL under the supervision of CS and JM. All authors contributed to the interpretation of the results. AL and SS drafted the manuscript, CS and JM provided critical revisions. All authors approved the final version of the manuscript for submission.

## Conflict of Interest Statement

The authors declare that the research was conducted in the absence of any commercial or financial relationships that could be construed as a potential conflict of interest.
